# The impact of COVID-19 on health status of home-dwelling elderly patients with dementia in East Lombardy, Italy: results from COVIDEM network

**DOI:** 10.1007/s40520-020-01676-z

**Published:** 2020-09-12

**Authors:** Salvatore Caratozzolo, Alberto Zucchelli, Marinella Turla, Maria Sofia Cotelli, Sara Fascendini, Mara Zanni, Angelo Bianchetti, Matteo Peli Psy, Renzo Rozzini, Stefano Boffelli, Melania Cappuccio, Federica Gottardi Psy, Chiara Vecchi Psy, Daniele Bellandi, Claudia Caminati, Simona Gentile, Elena Lucchi Psy, Ignazio Di Fazio, Marina Zanetti Psy, Giuliana Vezzadini, Chiara Forlani Psy, Maura Cosseddu Psy, Rosanna Turrone Psy, Silvia Pelizzari Psy, Andrea Scalvini, Marco Di Cesare, Marta Grigolo Psy, Lina Falanga, Nives Medici, Nives Palamini, Elisa Zanacchi Psy, Eleonora Grossi Psy, Giuseppe Bellelli, Alessandra Marengoni, Marco Trabucchi, Alessandro Padovani, On behalf of COVIDEM Network

**Affiliations:** 1 Neurology Unit, Department of Clinical and Experimental Sciences, Center for Neurodegenerative Disorders, Spedali Civili di Brescia, University of Brescia, Brescia, Italy; 2 Unità Geriatria, Dipartimento Scienze Cliniche e Sperimentale, Università degli Studi di Brescia, Brescia, Italy; 3 Neurology Unit, Azienda Socio-Sanitaria Territoriale della Valcamonica, Esine (Brescia), Italy; 4 Centro di Eccellenza Alzheimer FERB Onlus, Ospedale Briolini di Gazzaniga, Gazzaniga (Bergamo), Italy; 5 Gruppo San Donato – Ospedale Sant’Anna Brescia, Brescia, Italy; 6 Unità di Cure Sub Acute, Dipartimento di Geriatria, Fondazione Poliambulanza di Brescia, Brescia, Italy; 7 IPS Cardinal Gusmini, Vertova (Bergamo), Italy; 8 Coordinamento dei Caffè Alzheimer della Lombardia Orientale, Brescia, Italy; 9 Fondazione Sospiro, Cremona, Italy; 10 Fondazione Teresa Camplani - Casa di Cura Ancelle della Carità, Cremona, Italy; 11 Ospedale Richiedei, Palazzolo (Brescia), Italy; 12 IRCCS Maugeri, Castel Goffredo (Mantova), Italy; 13 Dipartimento Medicina e Chirurgia, Clinica Geriatrica, Università Bicocca, Milan, Italy; 14 Gruppo di Ricerca Geriatrica, Brescia, Italy

**Keywords:** COVID-19, Dementia, Home dwelling, Health status

## Abstract

**Background:**

COVID-19 outbreak has led to severe health burden in the elderly. Age, morbidity and dementia have been associated with adverse outcome.

**Aims:**

To evaluate the impact of COVID-19 on health status in home-dwelling patients.

**Methods:**

848 home-dwelling outpatients with dementia contacted from April 27 to 30 and evaluated by a semi-structured interview to evaluate possible health complication due to COVID-19 from February 21 to April 30. Age, sex, education, clinical characteristics (including diagnosis of dementia) and flu vaccination history were obtained from previous medical records. Items regarding change in health status and outcome since the onset of the outbreak were collected. COVID-19 was diagnosed in patients who developed symptoms according to WHO criteria or tested positive at nasal/throat swab if hospitalized. Unplanned hospitalization, institutionalization and mortality were recorded.

**Results:**

Patients were 79.7 years old (SD 7.1) and 63.1% were females. Ninety-five (11.2%) patients developed COVID-19-like symptoms. Non COVID-19 and COVID-19 patients differed for frequency of diabetes (18.5% vs. 37.9%, p < 0.001), COPD (7.3% vs. 18.9%, *p* < 0.001), and previous flu vaccination (56.7% vs. 37.9%, *p* < 0.001). Diabetes and COPD were positively associated with COVID-19, whereas higher dementia severity and flu vaccination showed an inverse association. Among COVID-19 patients, 42 (44.2%) were hospitalized while 32 (33.7%) died. Non COVID-19 patients’ hospitalization and mortality rate were 1.9% and 1.2%, respectively. COVID-19 and COPD were significantly associated with the rate of mortality.

**Discussion/conclusions:**

A high proportion of adverse outcome related to COVID-19 was observed in home-dwelling elderly patients with dementia. Active monitoring though telehealth programs would be useful particularly for those at highest risk of developing COVID-19 and its adverse outcomes.

**Electronic supplementary material:**

The online version of this article (10.1007/s40520-020-01676-z) contains supplementary material, which is available to authorized users.

## Introduction

The outbreak of coronavirus disease 2019 (COVID-19) hit Italy by the end of February and rapidly spread from Lombardy to the rest of the country, with a number of fatalities beyond 31,000. The COVID-19 outbreak is currently leading to severe health burden, in particular in Lombardy, the region with the highest number of infected people, highest number of hospitalization and deaths [1].

Although all regions reported having patients with COVID-19, the highest number of identified cases was in the provinces of eastern Lombardy, including Bergamo, Brescia and Cremona. Case-fatality rate steeply increases with age worldwide, where half of the deaths occurred in people aged 80 years or above [2, 3]. Dementia is highly prevalent among very old people (i.e., octogenarians) and is therefore expected to be particularly common among people who had the highest case-fatality rate related to COVID-19. Nonetheless, only a few studies have focused on the impact of COVID-19 on dementia. Importantly, demented subjects might be at higher risk of adverse events because they are generally old, are affected by multiple morbidities, have difficulty recognizing signs of infection, have limited access to information about COVID-19, and have difficulties in remembering safeguard procedures, thus ignoring the warnings and being exposed to higher chance of infection [4, 5]. As far as Italy is concerned, due to the outbreak, most outpatients’ clinics, including the Center of Dementia and Cognitive Decline (CDCD), were closed after the suspension of deferred outpatient visits and the extension of treatment plans according to the regional decrees. Patients and their caregivers were neither included in national health care programs or in regional health initiatives for active monitoring by telemedicine. Nevertheless, to accomplish patients’ needs and clinical issues, in several CDCD of the provinces of the Eastern Lombardy initiatives for remote listening, tele-consultation and telemedicine have been carried out. These initiatives have allowed maintaining in many cases a contact with the majority of patients followed up by the CDCD. Accordingly, most of these centres agreed to record clinical data routinely obtained through an interview in outpatients with cognitive decline or dementia.

The aim of this study was to evaluate the impact of the COVID-19 outbreak on the health status of a large sample of home-dwelling elderly with dementing illness, by investigating the rate of COVID-19 symptoms, rate of hospitalization and fatality. Clinical characteristics of patients from medical records and COVID-19-related prognostic factors were evaluated.

### Participants and procedure

This is an observational study carried out in home-dwelling patients with dementia who were periodically visited at the CDCD. These are specialist centres in connection with general practitioners (GPs), which have been established across the country and are dedicated to the diagnosis and management of patients for all forms of dementia across the entire complex staging spectrum. The sample included outpatients with a complete medical record before the beginning of the outbreak in Italy (February 21). Eleven CDCDs from Eastern Lombardy were selected according to their involvement in teleconsulting activities and accepted to participate and to perform a structured interview during the last week of April (27–30 April 2020). The interview (Supplementary Material) was carried out by a physician and a psychologist and was part of the routine evaluation to assist and care patients during the suspension of outpatient clinic. In case of moderate to severe cognitive impairment or severe COVID-19 disease, the interview was administered both to patients and proxy/caregiver. All data were anonymously aggregated.

The Ethical Committee of the Brescia County approved the study.

### Data collection

Information on age, sex, education, clinical characteristics including dementia diagnosis, dementia severity by Clinical Dementia Rating scale (CDR) [6], Mini Mental State Examination (MMSE) [7], and Basic Activity of Daily Life (BADL) [8], walking, total number of chronic diseases and type of diseases among a pre-defined list (hypertension, COPD, renal disease, heart disease, cancer, gastrointestinal diseases, hepatic disease, diabetes, thyroid disorders, arthritis), number of drugs, and flu vaccination were obtained from previous medical records based on the last recorded visit at CDCD. The semi-structured interview included items regarding change in health status, unplanned hospitalization and mortality in the previous 2 months, thus since the onset of the Italian outbreak (February 21). About the health status, symptoms and signs of possible COVID-19 infection (fever, cough, headache, dyspnoea, weakness, gastrointestinal problems) were collected. Patients were classified as affected by COVID-19, either if they had a positive swab test or a suspected infection according to WHO definition [9]. In particular, patients were considered to be affected by COVID-19 (a) if patients had a referred acute respiratory illness or COVID-like infection (fever and at least one sign/symptom of respiratory disease, such as cough, shortness of breath, dyspnoea, sore throat), AND with no other aetiology that fully explains the clinical presentation AND either a history of travel to or residence in a country/area or territory reporting local transmission of COVID-19 disease during the 14 days prior to symptom onset; (b) if patients had a severe acute respiratory infection (fever and at least one symptom or sign of respiratory diseases such as coughing and difficulty breathing) that required hospitalization for severe acute respiratory infection (SARI), with no other aetiology that fully explains the clinical presentation.

### Statistical analysis

The study population’s characteristics were described using mean and standard deviation or absolute number and proportion. Differences between groups were explored using *t* tests or *χ*^2^ test, as appropriate. Odds ratios (ORs) and their 95% confidence intervals (95% CI) were obtained using multivariable logistic regression models testing factors associated with COVID-19 and mortality. Variables significant at univariate analysis or considered clinically important according to the outcome of interest were included in the multivariate models. An alpha level of 0.05 was considered to be statistically significant for the analyses. Data analyses were carried out using SPSS software (version 22.0) and R (version 4.0.0; R Core Team, Wien-Austria).

## Results

In total, 900 patients were contacted from ten CDCD. Among them, 26 patients were missing, 10 patients refused to participate, and 16 patients were unable to give reliable information. The final sample of the study included 848 patients. As shown in Table 1, patients were on average 79.7 years old (SD 7.1), 63.1% were females, and 59.7% had primary education. According to historical data referred to the last valid visit (mean time from last visit and interview was 16.5 SD 8.3 weeks), mean CDR was 1.5 (SD 0.9) and mean MMSE was 18.7 (SD 6.8). The mean number of chronic diseases was five (SD 2.8) and the most frequent were hypertension (71.3%), cardiovascular disease (35.1%), type 2 diabetes mellitus (20.6), cancer (16.3%) and chronic obstructive pulmonary disease (COPD) (8.5%). The mean number of drugs was 5.5 (SD 2.8). Flu vaccination was reported in 463 patients (54.6%). According to medical records, 144 (17%) were patients affected by mild cognitive impairment (MCI), 331 (39%) by Alzheimer disease, 202 (23.8%) by mixed dementia, and 171 (20.2%) by other dementing illnesses, including fronto-temporal dementia, Lewy-body dementia, and vascular dementia.

**Table 1:** Demographic and Clinical Characteristic of the whole sample and grouped by COVID-19 infection/non infection

Variable	Overall	nonCOVID-19	COVID-19	*p*
	*N *= 848	*N *= 753	*N *= 95	
Age (mean (SD))	79.7 (7.1)	79.8 (7.1)	79.2 (7.5)	NS
Women (%)	535 (63.1)	476 (63.2)	59 (62.1)	NS
Diagnosis (%)				
MCI (%)	144 (17.0)	127 (16.9)	17 (17.9)	NS
Alzheimer disease (%)	331 (39.0)	303 (40.2)	28 (29.5)	NS
Mixed (%)	202 (23.8)	179 (23.8)	23 (24.2)	NS
Other (%)	171 (20.2)	91 (12.1)	16 (16.8)	NS
CDR (SD)	1.5 (0.9)	1.6 (0.9)	1.3 (0.9)	0.017
CDR 0.5 (%)	230 (27.1)	197 (26.2)	33 (34.7)	
CDR 1 (%)	198 (23.3)	172 (22.8)	26 (27.4)	
CDR 2 (%)	255 (30.1)	232 (30.8)	23 (24.2)	
CDR 3 (%)	165 (19.5)	152 (20.2)	13 (13.7)	
MMSE (SD)	18.7 (6.8)	18.6 (6.9)	20.5 (5.8)	0.010
BADL Lost (SD)	1.9 (2.1)	2.0 (2.1)	1.6 (1.9)	NS
Number of Diseases (SD)	5.0 (2.8)	5.1 (2.8)	4.7 (2.8)	NS
Number of Drugs (SD)	5.5 (2.9)	5.5 (2.8)	5.3 (2.8)	NS
Hypertension (%)	605 (71.3)	544 (72.2)	61 (64.2)	NS
Diabetes Mellitus (%)	175 (20.6)	139 (18.5)	36 (37.9)	< 0.001
COPD (%)	72 (8.5)	55 (7.3)	18 (18.9)	< 0.001
Heart Diseases (%)	298 (35.1)	265 (35.2)	33 (34.7)	NS
Liver Disease (%)	73 (8.6)	65 (8.6)	9 (9.5)	NS
Kidney Disease (%)	79 (9.3)	65 (8.6)	14 (14.7)	NS
Flu Vaccination (%)	462 (54.5)	427 (56.7)	36 (37.9)	0.001
Hospitalisation (%)	57 (6.7)	15 (2.0)	42 (44.2)	< 0.001
Institutionalization (%)	8 (0.9)	1 (0.1)	7 (7.4)	< 0.001
Death (%)	41 (4.8)	9 (1.2)	32 (33.7)	< 0.001

Ninety-five (11.2%) patients developed COVID-19 symptoms. Nasopharyngeal swab was performed in the whole sample of 57 hospitalized subjects. Forty-seven subjects were classified as COVID-19 positive only on suggestive symptoms with epidemiologic criteria; 16 patients with SARI symptoms were hospitalized; of those, 12 were positive at nasopharyngeal swab, while 4 patients were negative; 7 patients remained at home and were not investigated.

NonCOVID-19 and COVID-19 patients differed in CDR (1.6 SD 0.9 vs. 1.3 SD 0.9; *p *= 0.017), MMSE (18.6 SD 6.9 vs. 20.5 SD 5,8 *p *= 0.01), frequency of diabetes (18.5% vs. 37.9%, *p* < 0.001), COPD (7.3% vs. 18.9%, *p* < 0.001), and previous flu vaccination (56.7% vs. 37.9%, *p* < 0.001), while the two groups were comparable for age, gender, number of diseases, number of drugs, and BADL.

As shown in Table 2, diabetes (OR 3.1; CI 1.92–5.11) and COPD (OR 2.8; CI 1.51–5.16) were significantly associated with COVID-19 infection, whereas hypertension (OR 0.6; CI 0.35–0.94), moderate to severe dementia (OR 0.49, CI 0.27–0.91) and flu vaccination (OR 0.47; CI 0.29–0.74) were negatively associated with COVID-19 infection.

**Table 2:** Odds ratios (OR) and 95% confidence intervals (95% CI) for having COVID-19

Factor	OR	95% CI
Age (years)	0.9	0.96–1.02
Sex, male	1.1	0.69–1.75
Hypertension	0.6	0.35–0.94
Diabetes mellitus	3.1	1.92–5.11
COPD	2.8	1.51–5.16
CDR		
0.5	1 (reference)	–
1	0.8	0.46–1.53
2	0.49	0.27–0.91
3	0.49	0.24–1.01
Flu vaccination	0.47	0.29–0.74

*COPD* chronic obstructive pulmonary disease, *CDR* clinical dementia rating scale

**Fig. 1:**
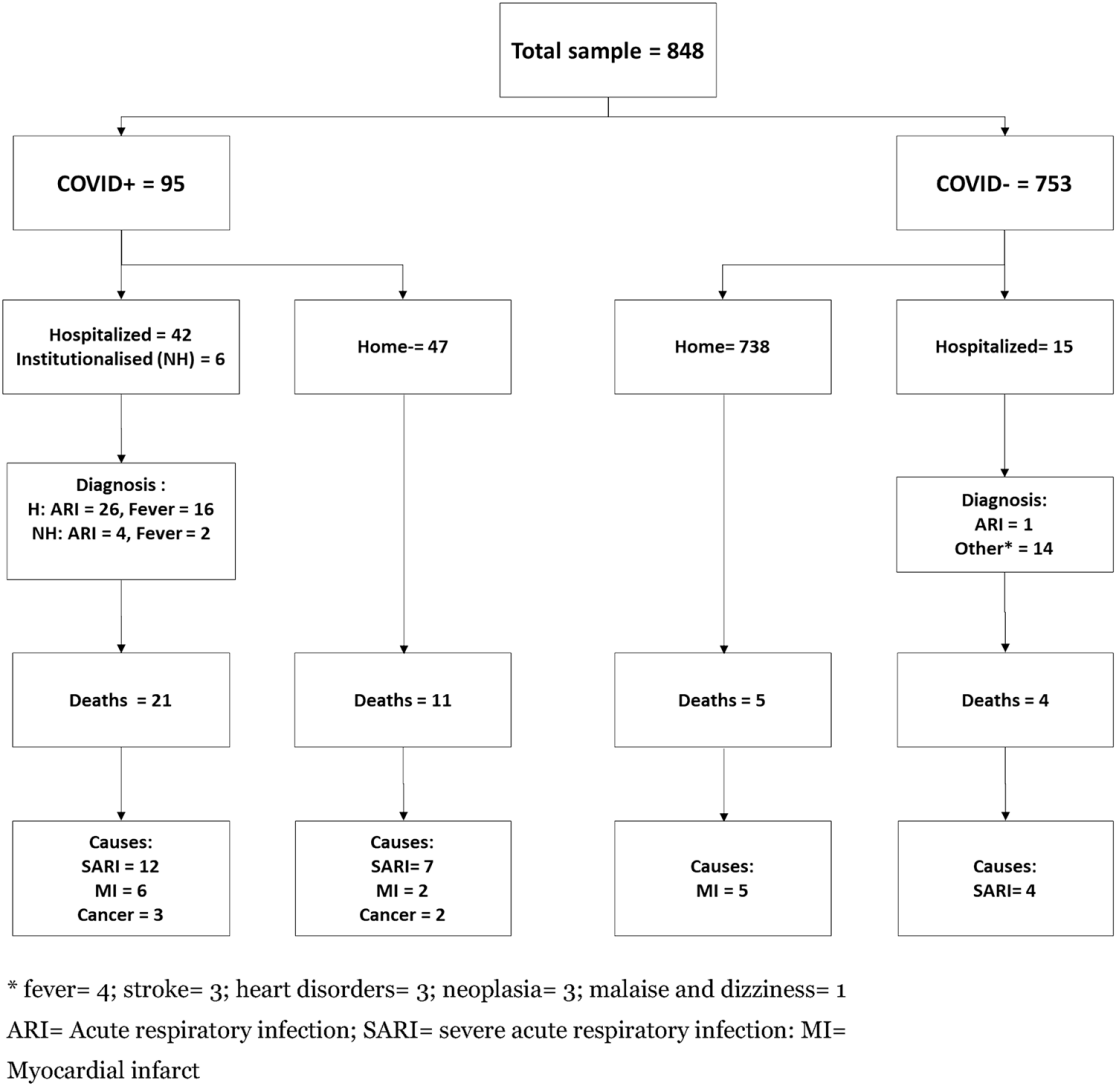
Flowchart of the study population

As reported in the flowchart of the study (Fig. 1), 42 (44.2%) COVID-19 patients were hospitalized whereas 6 COVID-19 patients were placed in a nursing home. None of non COVID-19 patients was institutionalized during the observation period. Thirty-two (33.7%) COVID-19 patients died (at home = 11; in hospital = 21) including 19 patients for SARI, 8 patients for myocardial infection and 5 patients for cancer. Among the rest of the sample (*n *= 753), 15 (1.9%) patients were hospitalized whereas 9 (1.2%) patients died at home (*n *= 5) or during hospitalization (*n* = 4).

As shown in Table 3, compared to survivors, patients who died showed milder dementia severity according to CDR and MMSE, but not age, gender, number of diseases and drug differences, whereas they differed in terms of frequency of hypertension, COPD and COVID symptoms.

**Table 3:** Demographic and clinical characteristics of the sample according to fatality

Variables	Survivors	Not Survivors	*p*
	*N *= 807	*n *= 41	
Age (mean (SD))	79.7 (7.1)	80.4 (7.3)	NS
Women (%)	506 (62.7)	29 (70.7)	NS
CDR (SD)	1.5 (0.9)	1.1 (0.7)	0.001
CDR 0.5 (%)	214 (26.5)	16 (39.0)	
CDR 1 (%)	185 (22.9)	13 (31.7)	
CDR 2 (%)	245 (30.4)	10 (24.4)	
CDR 3 (%)	163 (20.2)	2 (4.9)	
MMSE (SD)	18.6 (6.8)	21.4 (5.4)	0.011
BADL lost (SD)	1.9 (2.1)	1.3 (1.8)	0.034
Number of diseases (SD)	5.1 (2.8)	4.2 (2.3)	NS
Number of drugs (SD)	5.5 (2.8)	4.8 (2.8)	NS
Hypertension (%)	595 (73.3)	11 (26.9)	< 0.001
Diabetes mellitus (%)	165 (20.4)	10 (24.4)	NS
COPD (%)	65 (8.1)	8 (19.5)	0.019
Heart diseases (%)	288 (35.7)	9 (22.0)	NS
Liver disease (%)	72 (8.9)	2 (4.9)	NS
Kidney disease (%)	77 (9.5)	2 (4.9)	NS
Flu vaccination (%)	446 (55.3)	17 (41.5)	NS
COVID infection (%)	63 (7.8)	32 (78)	< 0.001
Hospitalisation (%)	37 (4.6)	20 (48.8)	< 0.001

In multivariate model testing factors associated with mortality, only COVID-19 symptoms (OR 94.5; 95% CI 31.0–287.9) and COPD (OR 3.7; 95% CI 1.21–11.43) were significantly associated.

## Discussion

Findings from our study showed that COVID-19 affected over 10% of home-dwelling older patients with dementia who showed high risk of adverse outcomes, such as unplanned hospitalization and mortality. COVID-19 was more frequently reported in patients with diabetes and COPD, whereas hypertension, dementia severity and anti-flu vaccination were significantly associated with lower likelihood of COVID-19. Further, COVID-19 and COPD were strongly associated with mortality. To the best of our knowledge, this is the first study that investigated the impact of COVID-19 on the health status of home-dwelling patients with dementia.

We used data collected during this emergency period to identify home-dwelling patients with dementia who developed COVID-19 and to test the hypothesis that they were at high risk of suffering an adverse outcome [2]. Findings of this study are in line with previous reports [10–12] and add new insight by showing that COVID-19 exerted a relevant impact on health status of home-dwelling elderly patients with dementia determining a high rate of hospitalization and mortality.

In this study, the proportion of patients that developed COVID-19 was close to the rates estimated [13, 14] in Lombardy (about 13%). In fact, in our survey 11.2% of the sample reported COVID-19 symptoms during the outbreak. These data argue for the effectiveness of the lockdown in limiting COVID-19 spreading among home-dwelling elderly, though asymptomatic cases and atypical cases might have been not properly identified, thus cases might be underestimated [15, 16]. In fact, it has been shown that in older and frail individuals such as those with dementia, infections may present without fever, cough, chest discomfort or other signs of inflammation [17, 18].

Previous findings have underlined that several factors are associated with COVID-19 including older age, metabolic and cardiovascular disease, and COPD [19–24]. In this study, COVID-19 was more frequent in patients with diabetes and COPD, whereas neither age nor gender predicted COVID-19 symptoms and adverse events. These findings are likely due both to the old age of the sample and to multiple morbidity, whereas the apparent protective effect of hypertension on COVID-19 might be due to a possible role of antihypertensive medications [25, 26]. As the study design does not allow to draw any conclusion, further studies are needed to disentangle the complex relationship between hypertension and risk COVID-19. At variance of literature data [4, 5], patients with advanced dementing illnesses were at lower risk to suffer COVID-19 and had less adverse events. There are several speculative explanations for this finding. First, in more advanced patients, preventive measures such as isolation and social distance might have been more strictly applied or, likely, patients with advanced dementia are those who were already confined at home since the beginning of the outbreak given their poor functional status. Secondly, we cannot rule out that patients with severe dementia had typical or atypical symptoms not recognized by caregivers as possibly associated with COVID-19 and thus not reported during the interview.

An important finding of the study is that flu vaccination was significantly associated with a lower risk of developing COVID-19. Flu vaccination was reported in about 54% of this sample, which is very close to the national coverage (53,1%) in the elderly population reported by Italian “Istituto Superiore Sanità” (ISS) [27, 28]. No studies have looked so far at the effect of flu vaccination on COVID-19 incidence or severity among elderly individuals. These unexpected results support the hypothesis that resultant immunity against prior influenza infection might, at least in part, foster immunity against SARS-CoV-2, due to the similarity in their structures and that flu vaccination might generate sustained bystander immunity that overall enhances immunity against SARS-CoV-2, thus inducing milder forms of COVID-19 [29, 30]. However, although we found a reduced odds of COVID-19 due to flu vaccination, our study was not specifically designed to address this research question; in fact, we did not account for timing of administration or type of vaccine received, nor did we address other potential factors that may have biased our results [31]. In fact, patients or caregivers who get a vaccination usually pay more attention to their health status and may be more likely to have been compliant with preventive measures. Not surprisingly, the prognosis of COVID-19 among this sample of home-dwelling patients was poor, as almost half of the affected patients were hospitalized, while among those who did not develop COVID-19 hospitalization was less than 2%, in line with epidemiological data [32]. Moreover, overall mortality rate was 33,7% among COVID-19 patients and less than 2% among non-COVID-19 patients. These data confirm recent reports in the Italian population which have shown that, in front of an overall fatality rate about 7%, the case-fatality rate of elderly patients is very high (i.e. 24% for decade 70–79 and 29% decade 80–89%) [33, 34]. Further, our data strongly support the claim that patients with dementing illnesses are even at higher risk of adverse events due to COVID-19 [35] and that they require careful evaluation and specific interventions early in the progression of the COVID-19 symptoms. To accomplish such a preventive intervention, national programs aimed to maintain a constant monitoring of elderly with dementing illnesses and at higher risk of developing COVID-19 diseases should be developed through a standardized protocol by telemedicine.

First, only a minority of patients included in the study were specifically tested for SARS-CoV-2 infection, whereas the majority of those classified as having COVID-19 exhibited symptoms considered COVID-19-like according to the definition of the WHO. On the other hand, given the outbreak of COVID-19 in Lombardy during the time period considered and that a late-seasonal flu was unlikely, the likelihood of a correct diagnosis of COVID-19 in patients included is high. Secondly, we cannot exclude that during the severe and unexpected public health emergency, the most severely affected patients were not even admitted to hospitals and died at home undiagnosed.

Under this assumption, our results are underestimating the impact of COVID-19, but this hypothesis should be tested in future studies.

On the other hand, the major strengths of the study are the few missing data and the novelty of clinical characteristics analysed.

## Conclusions

A high proportion of severe to critical cases of COVID-19 was observed in this large sample of home-dwelling elderly patients with dementia. Several patients’ conditions including diabetes, COPD, less severe dementing illness, and flu vaccination were associated with the risk of COVID-19. Close tele-monitoring and timely treatment of infection should be carried out as standardized care for home-dwelling elderly patients at high risk to prevent adverse events.

## Electronic supplementary material

Below is the link to the electronic supplementary material.

Supplementary material 1 (DOCX 17 kb)

## Data Availability

The raw data supporting the conclusions of this article will be made available by the authors, without undue reservation.
