# Supplementary material for: The impact of COVID-19 on health status of home-dwelling elderly patients with dementia in East Lombardy, Italy: results from COVIDEM network
Source: Aging Clin Exp Res. 2020 Sep 12;32(10):2133–40. doi: 10.1007/s40520-020-01676-z (PMC7486591; doi:10.1007/s40520-020-01676-z)
Supplement: Supplementary file 1 — Supplementary material 1 (DOCX 17 kb) [file 40520_2020_1676_MOESM1_ESM.docx]

Supplementary Materials

Questionnaire for the detection of the health conditions of patients belonging to the CDCD of the Provinces of Brescia, Cremona, Bergamo

GENERAL INFORMATIONS

| Date |  |  |
| --- | --- | --- |
| Name of the examiner |  |  |
| Initials Patient |  |  |
| Patient Code  ( Patient Initials, Examiner Initials, Reference Province, Patient Birth Year (e.g. Lsapbs1961 ) | Aabbccnnnn |  |
| Patient Residence |  |  |
| Age |  |  |
| Cohabitants Number |  |  |

PATIENT INFORMATION (REPORT FROM ARCHIVE AS KNOWN TO THE LAST VISIT)

| Last visit date CDCD | ddmmyyyy |  |
| --- | --- | --- |
| Diagnosis | MCI  AD  FTD  DLB  Mixed dementia  Vascular dementia  Other |  |
| CDR | 0.5  1  2  3  > 3 |  |
| MMSE (last check) raw score |  |  |
| BADL Lost |  |  |
| Diseases number |  |  |
| Number of drugs |  |  |
| Somatic DIseases | Hypertension  Diabetes  COPD  Heart disease  Liver disease  Kidney disease  Neoplasia  Arthritis | Yes No  Yes No  Yes No  Yes No  Yes No  Yes No  Yes No  Yes No |
| Influenza vaccination  If the data is missing, this should be requested during the telephone contact |  | Yes No |

QUESTIONNAIRE (information to be obtained from the patient or caregiver with reference to the patient). The examiner must introduce himself, declare the purpose of this interview, inquire about the general condition of the patient, or if the patient is still at home, in another home, in RSA , or hospitalized or deceased.The FIRST QUESTION refers to the caregiver. Degree of relativeness of the respondent: spouse, son or daughter, nephew, cousin, other relatives, other

Do you live with the patient? Yes No

Therefore the SECOND QUESTION must necessarily concern whether the patient is still alive.

" We would like to know if Mr. / Mrs. had health problems and whether he died because of them"  Yes no

If the patient has died, you will go directly to the death questionnaire.

Otherwise, the following information relating to where the patient is located will be requested.

Domiciled at home  Yes no

Domiciled to another family member  Yes no

Admitted to NURSING HOME  Yes no

Hospitalized  Yes no

Deceased  Yes no

In the event of DEATH, condolences being made, little information will be requested

| Date of death | mmddyyyy |  |
| --- | --- | --- |
| Reason for death | to specify | ________________________________ |
| Site of death | Home  Hospital  Rehabilitation  NHome | Yes No  Yes No  Yes No  Yes No |
| Recent history of fever |  | Yes No  Yes No |
| COVID diagnosis |  | Yes No |

In the event that the patient has been RECOVERED, the examiner will request the following information :

| Hospital | name |  |
| --- | --- | --- |
| Department Unit | name |  |
| Reason For Hospitalization | fever  Pneumonia  Other | Yes No  Yes No  To specify_________________________ |
| Hospitalization Date |  |  |
| If admitted to ICU |  | Yes No |
| If intubated |  | Yes No |
| Covid Diagnosis |  | Yes No |

In the event that the patient is at home or at another home or at RSA

| Recent history of deterioration | Yes No | Period of onset  > 2 months  > 1 month  > 15 days  <15 days |
| --- | --- | --- |
| Recent history of worsening memory | Yes No | > 2 months  > 1 month  > 15 days  <15 days |
| Recent history of fever | Yes No | > 2 months  > 1 month  > 15 days  <15 days |
| Recent history of sore throat | Yes No | > 2 months  > 1 month  > 15 days  <15 days |
| Recent history of cough | Yes No | > 2 months  > 1 month  > 15 days  <15 days |
| History of breathlessness or fatigue | Yes No | > 2 months  > 1 month  > 15 days  <15 days |
| History of muscle pain | Yes No | > 2 months  > 1 month  > 15 days  <15 days |
| History of gastrointestinal disorders | Yes No | > 2 months  > 1 month  > 15 days  <15 days |
| History of confusion and dizziness | Yes No | > 2 months  > 1 month  > 15 days  <15 days |
| History of instability and / or difficulty of the march | Yes No | > 2 months  > 1 month  > 15 days  <15 days |
| History of deterioration of functional capacity |  |  |
| History of behavior disorders | Depression  Insomnia  agitation  Wandering  Hallucinations | Yes No  Yes No  Yes No  Yes No  Yes No |
| Other ailments | Yes No | To specify ________________________________ |
| Contact with General Practitioner | Yes No | > 2 months  > 1 month  > 15 days  <15 days |
| Diagnosis of COVID BY SWAB | Yes No | Date _________________________ |
